# Metagenomic analysis of viral community in the Yangtze River expands known eukaryotic and prokaryotic virus diversity in freshwater

**DOI:** 10.1016/j.virs.2022.01.003

**Published:** 2022-01-13

**Authors:** Juan Lu, Shixing Yang, Xiaodan Zhang, Xiangming Tang, Ju Zhang, Xiaochun Wang, Hao Wang, Quan Shen, Wen Zhang

**Affiliations:** a Department of Laboratory Medicine, School of Medicine, Jiangsu University, Zhenjiang, 212013, China; b Department of Clinical Laboratory, The Affiliated Huai'an Hospital of Xuzhou Medical University, Huai'an, 223002, China; c Zhenjiang Center for Disease Prevention and Control, Zhenjiang, 212000, China; d State Key Laboratory of Lake Science and Environment, Nanjing Institute of Geography and Limnology, Chinese Academy of Sciences, Nanjing, 210008, China

**Keywords:** Freshwater virome, River water, Viral metagenomics, Virus diversity

## Abstract

Viruses in aquatic ecosystems are characterized by extraordinary abundance and diversity. Thus far, there have been limited studies focused on viral communities in river water systems. Here, we investigated the virome of the Yangtze River Delta using viral metagenomic analysis. The compositions of viral communities from six sampling sites were analyzed and compared. By using library construction and next generation sequencing, contigs and singlet reads similar to viral sequences were classified into 17 viral families, including nine dsDNA viral families, four ssDNA viral families and four RNA viral families. Statistical analysis using Friedman test suggested that there was no significant difference among the six sampling sites (*P* ​> ​0.05). The viromes in this study were all dominated by the order *Caudovirales*, and a group of *Freshwater phage uvFW* species were particularly prevalent among all the samples. The virome from Nanjing presented a unique pattern of viral community composition with a relatively high abundance of family *Parvoviridae*. Phylogenetic analyses based on virus hallmark genes showed that the *Caudovirales* order and CRESS-DNA viruses presented high genetic diversity, while viruses in the *Microviridae* and *Parvoviridae* families and the *Riboviria* realm were relatively conservative. Our study provides the first insight into viral community composition in large river ecosystem, revealing the diversity and stability of river water virome, contributing to the proper utilization of freshwater resource.

## Highlights

• First insight into viral community composition in large river ecosystem.
• Virus hallmark genes present both diverse and conservative characteristics.
• The composition of viral communities is similar on the whole.
• Slight regional variation of virome is existed in individual areas.

## 1 Introduction

Aquatic environments possess highly abundant and diverse viruses, which are approximately an order of magnitude higher than the total number of cellular organisms (Bergh et al., 1989; Maranger and Bird, 1995; Suttle, 2007; Paez-Espino et al., 2016). Viruses can be regarded as one of the primary causes of morbidity and mortality for aquatic life (Lang et al., 2009). Vertebrate enteric viruses, such as adenoviruses, enteroviruses and rotaviruses (Chigor et al., 2014; Mackowiak et al., 2018; Sedji et al., 2018), are more likely to contaminate freshwater and transmitted to human or animals through drinking, swimming, or inhaling aerosol (Gall et al., 2015; Kistemann et al., 2016). Besides, Bacteriophages, which are dominant in aquatic ecosystems, are capable of modulating the composition and abundance of bacterial communities (Fischer and Velimirov, 2002), and further affecting the recirculation of nutrients and the occurrence of algal bloom (Paerl and Otten, 2013). Aquatic ecological environment, in turn, may further meditate intraspecific and interspecific transmission of viruses (Mehle and Ravnikar, 2012). For example, some tomato and potato viruses, such as pepino mosaic virus and potato virus Y, are very stable in the water and could efficiently spread via water media to infect healthy plants (Mehle et al., 2014). Thus, it is of great necessity to explore the structure of viral communities in aquatic environments so as to ensure the sustainable development of water resources.

Most previous research on water virome has focused on viruses in marine waters, revealing the constitution and distribution of marine viruses (Breitbart et al., 2002; Culley et al., 2006; Suttle, 2016; Gregory et al., 2019). Freshwater, as the main source of drinking water, only accounts for 2.5% of the total volume of water on earth, and meanwhile, the burgeoning population and changing climate put us in the midst of a global freshwater crisis (Harrison et al., 2018; Dudgeon, 2019). Previous research indicated that freshwater harbored specific viral communities which were distinct from other aquatic environments based on hierarchical cluster analysis (Roux et al., 2012; Kim et al., 2015). Other studies have described the characterization of freshwater viromes in lakes (Lopez-Bueno et al., 2009; Roux et al., 2012; Ge et al., 2013), ballast water (Kim et al., 2015), sewage (Fernandez-Cassi et al., 2018; Martinez-Puchol et al., 2020), etc. However, only a few studies have explored the viral communities in rivers, and most of them investigated viromes in river estuaries, where marine and freshwater mix (Cai et al., 2016; Wolf et al., 2020; Wu et al., 2020). A metagenomic investigation of a river in Spain revealed that most viral reads were assigned into plant-infecting families, and some human pathogenic viruses were also identified (Fernandez-Cassi et al., 2017). However, this river was only 17.7 ​km in length and was seriously contaminated by daily and industrial sewage; thus it might be an unrepresentative case. Therefore, it is very imperative to conduct investigations about viruses in river water systems. .

The Yangtze River, as the longest river in Asia (6300 ​km), rises in the Tanggula Mountains and empties into the East China Sea. Thereinto, the Yangtze River Delta region, located in the lower reaches of the Yangtze River, is one of the most densely populated regions on earth and the most economically developed area of China (Shao et al., 2019). Hence, the Delta region could be regarded as a typical sample for river water virome research. Till now, the composition of viral community in this region has remained unknown. Here, the aims of this study were to: (1) explore the virome characteristics in the largest river in Asia, (2) compare the viral communities among samples from different representative cities in the Delta region and (3) phylogenetically analyze the genetic diversity of main virus groups based on a series of virus hallmark genes. The results from this study could provide a typical distribution pattern of viruses in river water ecosystem, which may contribute to the rational use of scarce freshwater resources.

## 2 Material and methods

### 2.1 Sample collection and preparation

To investigate the freshwater virome from the Yangtze River, approximately 5 ​L of water samples were collected between 2017 and 2018 from each of the six representative river ports in the Yangtze River Delta: Anqing, Wuhu, Nanjing, Zhenjiang, Changzhou and Nantong. The sampling section is around 640 ​km in length, accounting for about one-tenth of the total length and basically covering the whole Yangtze River Delta region. The information of sampling sites was listed in Supplementary Table S1. The water samples were collected using the five-point sampling method at 20 ​cm water depths in each sampling site. The center of the river was determined as the central sampling point, and then four points on the diagonal apart from the central sampling point were selected. A total of 30 ​L of water samples were collected with sterile disposable containers and shipped on ice immediately for further processing. As a control, 5 ​L sterile ddH_2_O (Sangon, Shanghai, China) was simultaneously prepared and further processed in the same condition.

### 2.2 Viral particles concentration

Viral particles were concentrated using the virus adsorption-elution method reported by Katayama et al., (2002) and further optimized by Hamza et al., (2009) and De Keuckelaere et al. (De Keuckelaere et al., 2013). Briefly, in the initial primary concentration step, MgCl_2_ was added to the water samples to obtain a final concentration of 0.05 ​mol/L, and the pH was adjusted to 3.5 with 1 ​mol/L HCl. Glass fiber filters (AP15 and AP20, Millipore) were used as prefilters to delay clogging. A type HA negatively charged membrane (HAWP14250, Millipore) with 0.45 ​μm pore size was used in a pressure pump system for water filtration. After filtration, the membranes were rinsed with 0.5 ​mmol/L H_2_SO_4_ (pH 3.4) and eluted with 70 ​mL Tr alk elution buffer (0.05 ​mol/L KH_2_PO_4_, 1.0 ​mol/L NaCl, 0.1% (v/v) Triton X-100, pH 9.2) (Hamza et al., 2009). In the secondary concentration step, 12.5% (w/v) PEG-6000 (Sigma-Aldrich) and 0.3 ​mol/L NaCl (Sigma-Aldrich) were added. After overnight incubation at 4 ​°C, the concentrate was centrifuged at 10,000×*g* for 30 ​min. Then the pellet was suspended in 2 ​mL PBS and vigorously vortexed. The mixture was incubated for 5 ​min at room temperature and centrifuged at 10,000×*g* for 20 ​min. About 2 ​mL supernatant was collected and stored at −80 ​°C until use.

### 2.3 Viral metagenomic library construction

The concentrated water samples were treated at 37 ​°C with a mixture of DNases (Turbo DNase from Ambion, Baseline-ZERO from Epicentre, and benzonase from Novagen) and RNase (Fermentas) for 60 ​min to digest unprotected nucleic acid (Zhang et al. 2014, 2016, 2017). Then the remaining total nucleic acid was isolated using a QIAamp Viral RNA Mini Kit (QIAGEN) according to the manufacturer's protocol. For library construction, dsDNA was synthesized from RNA and DNA viruses. For RNA viruses, a reverse transcription kit (SuperScript III Reverse Transcriptase) was used for reversely transcribing RNA into cDNA, after which the product was denatured at 95 ​°C for 2 ​min and quickly placed on ice for about 2 ​min. Then the DNA polymerase I large fragment (Klenow) was added to synthesize the second strand of cDNA (dsDNA). For ssDNA viruses, ssDNA was converted to dsDNA using the Klenow reaction and the product was utilized to construct libraries. Specifically, 12 ​μL nucleic acid extracts were added to the reaction system for synthesizing dsDNA (total reaction system: 20 ​μL) and the experiments were performed in the same tube. Overall, six libraries along with a control library were constructed using a Nextera XT DNA Sample Preparation Kit (Illumina) and the quality was inspected using agarose gel electrophoresis and Agilent bioanalyzer 2100. All libraries were sequenced on an Illumina MiSeq platform (250 bp paired ends) with dual barcoding for each individual sample (Liu et al., 2016).

### 2.4 Bioinformatics analysis

Paired-end reads of 250 bp generated by MiSeq were debarcoded using vendor software from Illumina. An in-house analysis pipeline running on a 32 nodes Linux cluster was utilized to process the data. Reads were considered duplicates if bases 5–55 were identical and only one random copy of duplicates was kept. Low sequencing quality tails were trimmed using Phred quality score ten as the threshold. Adaptors were trimmed using the default parameters of VecScreen which is NCBI BLASTn with specialized parameters designed for adapter removal. The cleaned reads were *de novo* assembled within each barcode, detected chimera are filtered by length using the ENSEMBLE assembler with the default parameters (Deng et al., 2015). Contigs and singlets reads are then matched against a customized viral proteome database using BLASTx with an E-value cutoff of <10^−5^. The virus BLASTx database was compiled using NCBI virus reference proteome (ftp://ftp.ncbi.nih.gov/refseq/release/viral/) and viral proteins sequences from NCBI nr fasta file (based on annotation taxonomy in Virus Kingdom). Candidate viral hits are then compared to an in-house non-virus non-redundant (NVNR) protein database with an E-value cutoff of <10^−5^ to remove false-positive viral hits. The NVNR database was compiled using non-viral protein sequences extracted from NCBI nr fasta file (based on annotation taxonomy excluding Virus Kingdom). Contigs without significant BLASTx similarity to viral proteome database are searched against viral protein families in vFam database (Skewes-Cox et al., 2014) using HMMER3 (Eddy, 2009; Johnson et al., 2010; Finn et al., 2011) to detect remote viral protein similarities.

### 2.5 Viral community analysis

Composition similarity analysis of the six viromes were compared using MEGAN software (MEtaGenome Analyzer, v6.20.19)(Huson et al., 2007) under the compare option. The results were presented by the Unweighted Pair Group Method with Arithmetic Mean (UPGMA) taxonomic tree, canonical correspondence analysis (CCA) under cluster analysis option, and Bray-Curtis ecological distance matrix with default parameters. The species rarefaction curve is also calculated and generated by MEGAN software v6.20.19 (Huson et al., 2007) in rarefaction window to evaluate sampling completion in each library. The Friedman rank-sum test was used for analyzing the differences of viromic structure among six viromes using SPSS (IBM SPSS 25.0, SPSS Inc)(Friedman, 1937). The viral community structure and richness results were visualized in heapmap, venn diagram and bar plots which were generated using R v3.6.3 package pheatmap (v1.0.12, https://cran.r-project.org/package=pheatmap), venn (v1.9, https://cran.r-project.org/package=venn) and ggplot2 (v3.2.1, https://ggplot2.tidyverse.org), respectively.

### 2.6 Viral sequences extension and annotation

Viral contigs that may be from the same genome but without overlaps were merged using the Low Sensitivity/Fastest parameter in software Geneious v11.1.2 (Kearse et al., 2012). And the individual contig was used as reference for mapping to the raw reads of its original barcode using the Low Sensitivity/Fastest parameter. Putative viral open reading frames (ORFs) were predicted by Geneious v11.1.2 with built-in parameters (Minimum size: 300; Genetic code: Standard; Start codons: ATG) (Kearse et al., 2012), further the predicted ORFs were compared against the nr database from NCBI using BLASTp. The annotations of these ORFs were based on comparisons to the Conserved Domain Database using RPS-BLAST with an E-value cutoff of <10^−5^. Coding protein sequences from ORFs which had no significant similarity found in the Database were annotated as putative proteins. Those contigs annotated with virus hallmark genes of main virus groups were selected, among which complete ORFs identified were included for further phylogenetic analyses (virus hallmark genes used: major capsid protein (MCP) for *Microviridae*, nonstructural protein 1 (NS1) for *Parvoviridae*, replication-associated protein (Rep) for CRESS-DNA viruses, TerL for *Caudovirales* and RNA dependent RNA polymerase (RdRp) for *Ribovriia*). All sequences with virus hallmark genes were presented in scatter plots drawn utilizing R package ggplot2 v3.2.1.

### 2.7 Phylogenetic analysis

Phylogenetic analyses were performed based on the predicted protein sequences of virus hallmark genes identified in this study and protein sequences of reference strains belonging to different group of viruses downloaded from the NCBI GenBank database. Related protein sequences were aligned using MUSCLE in MEGA v10.1.8 (Kumar et al., 2018) with the default settings. Sites containing more than 50% gaps were temporarily removed from alignments. Bayesian inference trees were then constructed using MrBayes v3.2.7 (Ronquist et al., 2012). The Markov chain was run for a maximum of one million generations, in which every 50 generations were sampled and the first 25% of Markov chain Monte Carlo (mcmc) samples were discarded as burn-in. Maximum Likelihood trees were also constructed to confirm all the Bayesian inference trees using software MEGA v10.1.8 (Kumar et al., 2018).

### 2.8 Quality control

Particular attention was given to minimizing the risk of cross contamination and nucleic acid degradation. Aerosol filter pipet tips were used for avoiding possible cross contamination among samples. All experimental materials (including microcentrifuge tubes, pipet tips, etc.) which directly contacted with nucleic acid samples were RNase and DNase free and all nucleic acid samples were dissolved in DEPC-treated water with RNase inhibitors added. All experimental processes were performed in a biological safety cabinet.

## 3 Results

### 3.1 Overview of sequencing outcomes

**Fig. 1:**
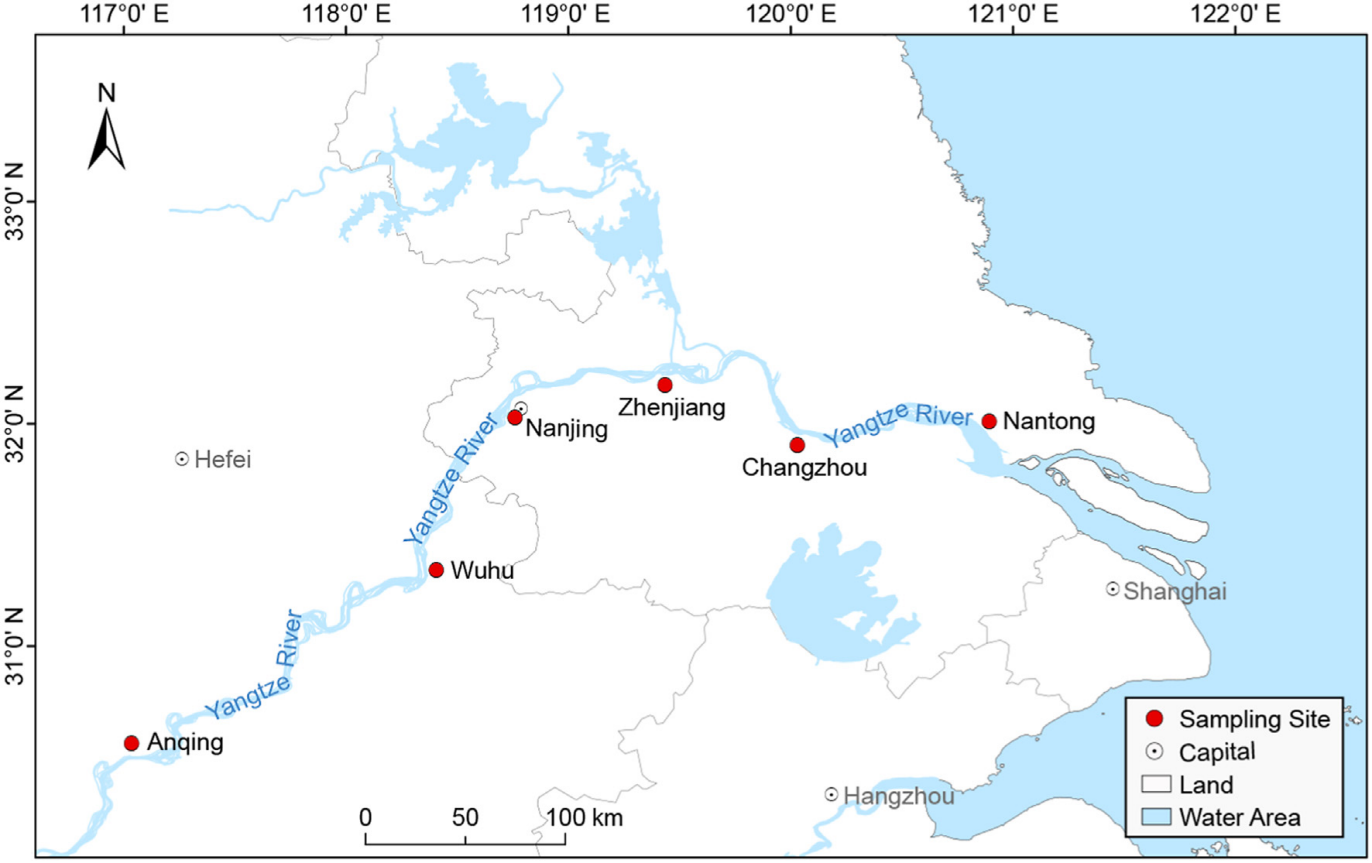
Map of the Yangtze River Delta with sampling sites. The sampling sites are indicated by red dots and labelled with city names.

To investigate viral communities in the Yangtze River, a complicated pipeline was established to collect freshwater samples from six river ports along the Yangtze River. As shown in Fig. 1, the sampling sites were distributed in Anhui Province (Anqing City and Wuhu City) and Jiangsu Province (Nanjing City, Zhenjiang City, Changzhou City, and Nantong City), respectively. After library construction and next generation sequencing on Illumina Miseq platform, the six freshwater libraries totally generated 6,454,680 raw reads with an average length of 235 bp and an average GC% of 51.8% (Supplementary Table S1). A total of 30,143 viral contigs (260 bp ​∼ ​20,778 bp) were obtained through *de novo* assembly and aligned against the viral protein database using BLASTx. And the percentage of raw reads mapped to the viral contigs in each library ranged from 10.7% to 26.0%. The control library generated 11,312 reads, accounting for around 1.05% of the average reads number in other libraries. The BLASTx searching based on the reads in control library revealed no viral sequences, indicating that the effect of cross-library contamination during the experimental processes is negligible.

### 3.2 Composition and comparison of viral communities

**Fig. 2:**
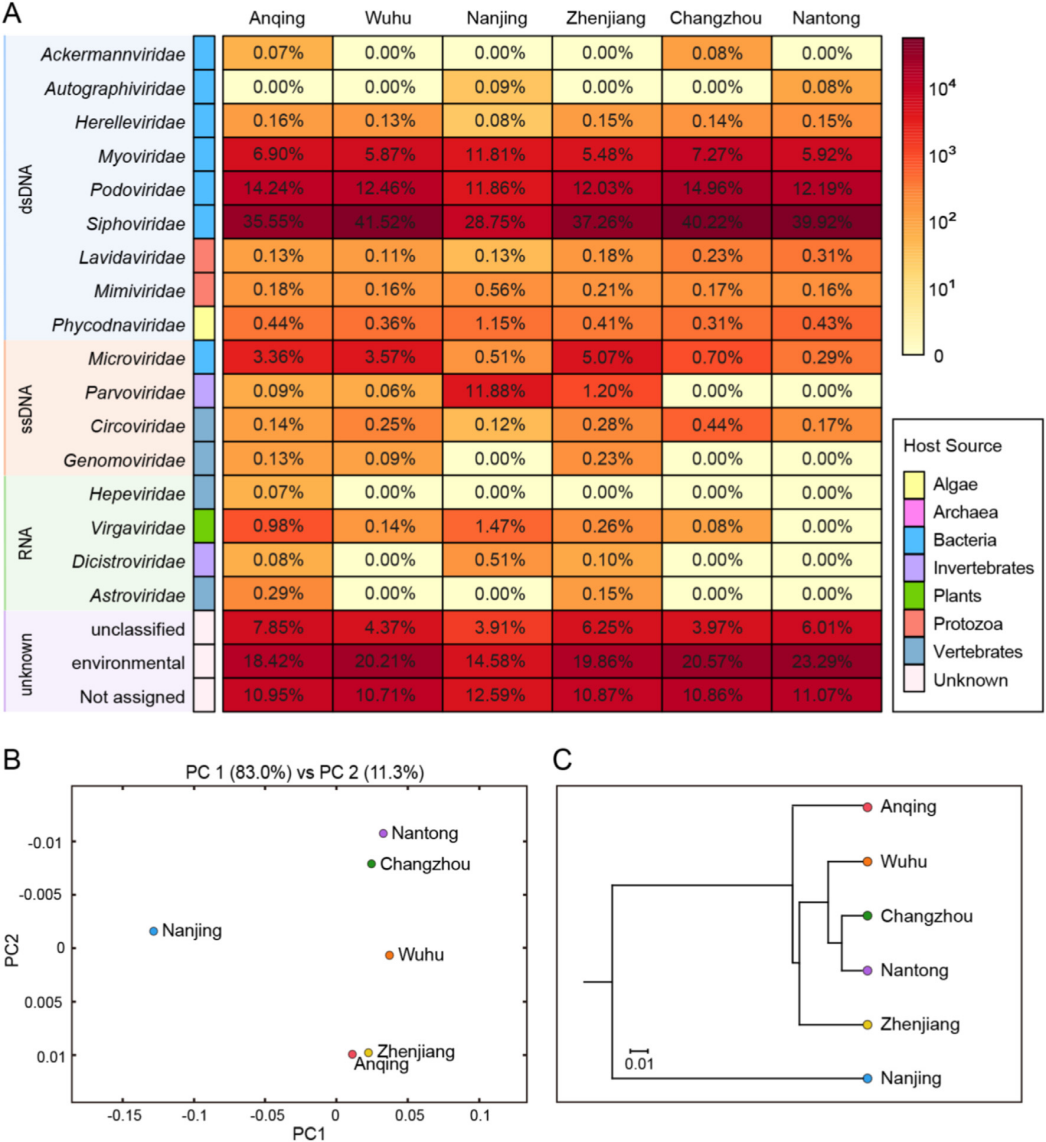
Taxonomic analyses of viral metagenomic reads on the family level. (A) Heatmap representing the reads number of each viral family in exponential form. Host sources are indicated on the left with the corresponding colors (see color legend). Different types of genome composition were represented by rectangles filled with different colors and taxon names are indicated on the left of rectangles. The percentages of each viral family in six sampling points were shown in the corresponding rectangles. **(B)** PCoA plot and **(C)** UPGMA taxonomic tree showing the similarity of viral community structures at each sampling site.

Contigs and singlets reads similar to viral sequences were classified into 17 viral families, including nine dsDNA viral families, four ssDNA viral families and four RNA viral families (Fig. 2A). Although the Friedman test suggested that there was no statistically significant difference among the samples (*P* ​> ​0.05), the heterogeneity between groups was observed. Most of the viral reads in the six libraries belong to the order *Caudovirales*, which was dominated by *Siphoviridae* (28.75%–41.52%), *Podoviridae* (11.86%–14.96%), *Myoviridae* (5.48%–11.81%) and other bacteriophage families. Relatively low numbers of sequences were classified into other dsDNA families, such as algae-infecting *Phycodnaviridae* (0.31%–1.15%), protist-infecting *Mimiviridae* (0.16%–0.56%) and its satellite viral family *Lavidaviridae* (0.11%–0.31%). The most predominant virus family of ssDNA viruses is also a bacteriophage family *Microviridae* (0.29%–5.07%)*.* The virome from Nanjing contains the predominant family *Parvoviridae* (11.88%) that mainly infects invertebrates. Other members of ssDNA group were identified to be homologous to CRESS-DNA viruses, which mainly consist of *Circoviridae* (0.12%–0.44%) and *Genomoviridae* (0.00%–0.23%) families that both infect a wide range of vertebrates. A small proportion of sequences were assigned to *Riboviria* realm including vertebrate-infecting *Astroviridae* (0.00%–0.29%) and *Hepeviridae* (0.00%–0.07%), invertebrate-infecting *Dicistroviridae* (0.00%–0.51%) and plant-infecting *Virgaviridae* (0.00%–1.47%) families, all of which were distributed sporadically in each of the six libraries. On average, 16.57% of viral reads were not classified or assigned into known viral families, and 19.49% of reads were similar with the sequences of uncultured viruses. Comparison analysis of the six viromes was carried out to evaluate the uniqueness and convergence among them. The principal coordinate composition (PCoA) analysis (Fig. 2B) and the UPGMA dendrogram (Fig. 2C) showed a clear separation between Nanjing and the other five sampling sites. Meanwhile, Nantong and Changzhou were clustered together closely and thus shared similar virome composition.

**Fig. 3:**
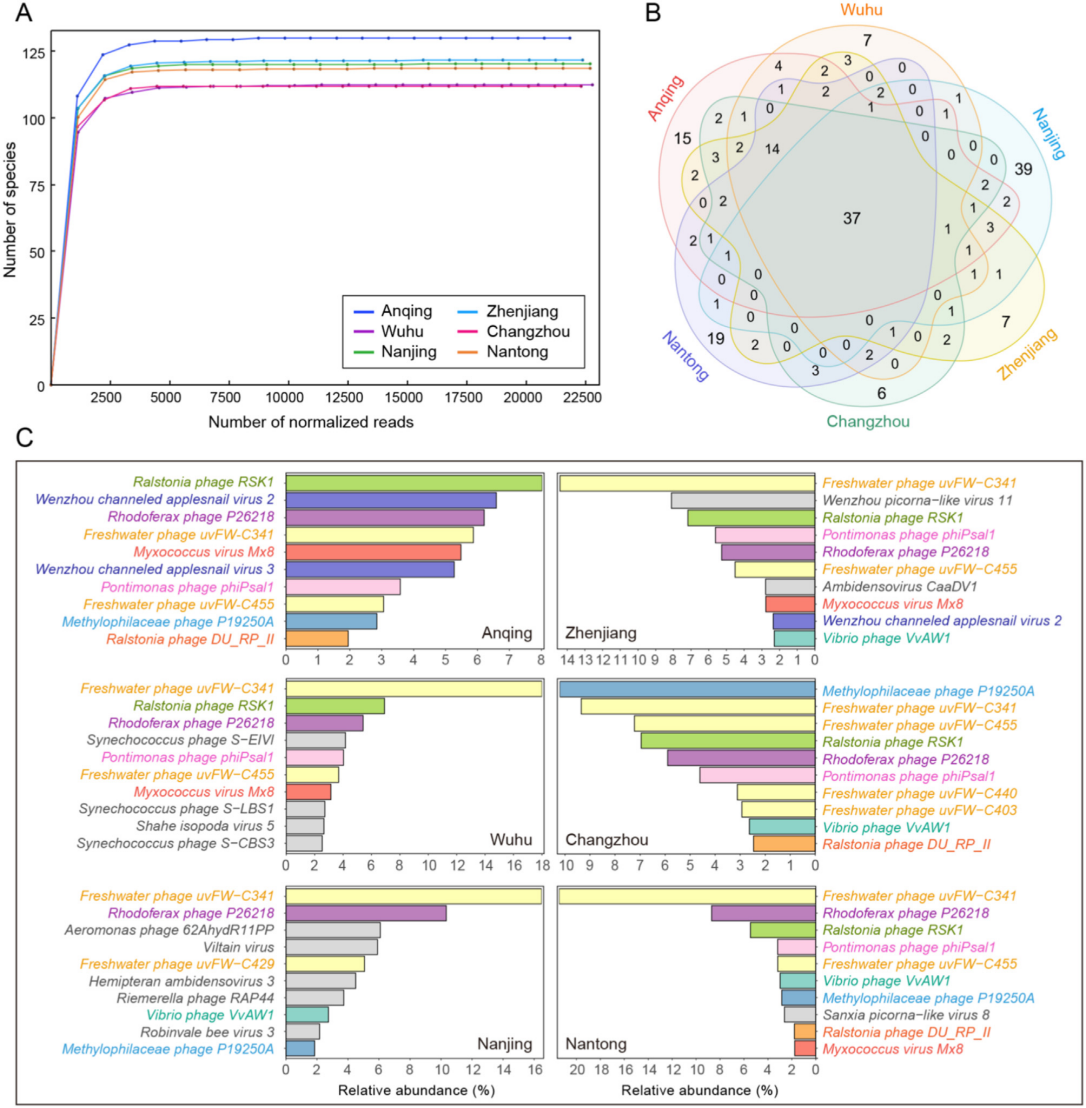
Taxonomic analyses of viral metagenomic reads on the species level. (A) Rarefaction curves of viral species in each sample. **(B)** Venn diagram depicting the distribution of shared and distinct viral species among the six viromes. **(C)** Bar plots showing the top 10 most abundant viral species in the six samples. The shared species among each virome are indicated with consistent color, and the specific species are indicated with gray. The horizontal axis indicates the relative abundance of reads assigned to each species and the scale of horizontal axes was adjusted in each panel.

Rarefaction curves of the six freshwater libraries yielded a horizontal asymptote, demonstrating that the sequencing depth might be sufficient to capture almost all known viral species in the samples and the sequencing data were rational and cogent (Fig. 3A). The venn diagram showed that a total of 201 viral species were detected in the six viral communities, among which we identified 37 overlapping species existing in all samples, accounting for 31%–45% of viral species found in each of the six samples. These results indicated that nearly half of the viral species were common in the six samples. However, it was apparent that some viral species were exclusive to an individual virome, revealing the viral diversity among different locations. For example, virome from Nanjing contained the highest number of unique species, accounting for 47% of the total (Fig. 3B). As for the viral species richness analysis, the top 10 most abundant viral species in each viral community were plotted in Fig. 3C, which were dominated by bacteriophages. A group of species *Freshwater phage uvFW* was shared among all the six libraries, accounting for a large proportion of viral species in each library, ranging from 8.93% to 24.63%; the second most abundant species was *Rhodoferax phage P26218,* ranging from 5.27% to 10.34%. Meanwhile, the unique species were also presented in each virome. For instance, the sample from Nanjing had the largest number of distinctive viral species, such as *Aeromonas phage 62AhydR11PP*, *Viltain virus* and *Hemipteran ambidensovirus 3*, demonstrating that Nanjing possessed a special viral community signature. In addition, the unique species in Wuhu mainly belong to *Synechococcus* phages, a group of bacteriophages infecting the phylum *Cyanobacteria* associated with the occurrence of bloom.

### 3.3 Identification of virus hallmark gene sequences

In this study, 1606 viral sequences were generated through sequence extension and annotation. All sequences were classified into five main groups according to reads abundance and conserved domains (i.e. virus hallmark genes (Koonin et al., 2006; Koonin et al., 2020)), including MCP for *Microviridae*, NS1 for *Parvoviridae*, Rep for CRESS-DNA viruses, phage terminase large subunit (TerL) for *Caudovirales* and RdRp domain for *Riboviria.* The BLASTx results showed that those sequences shared 23.62%–100% sequence identities, their length ranges from 274 bp to 10,160 bp with an average length of 1105 bp, and 301 sequences among them had complete coding sequences (CDS) of virus hallmark genes (Supplementary Fig. S1).

### 3.4 Phylogenetic analyses of viral sequences

**Fig. 4:**
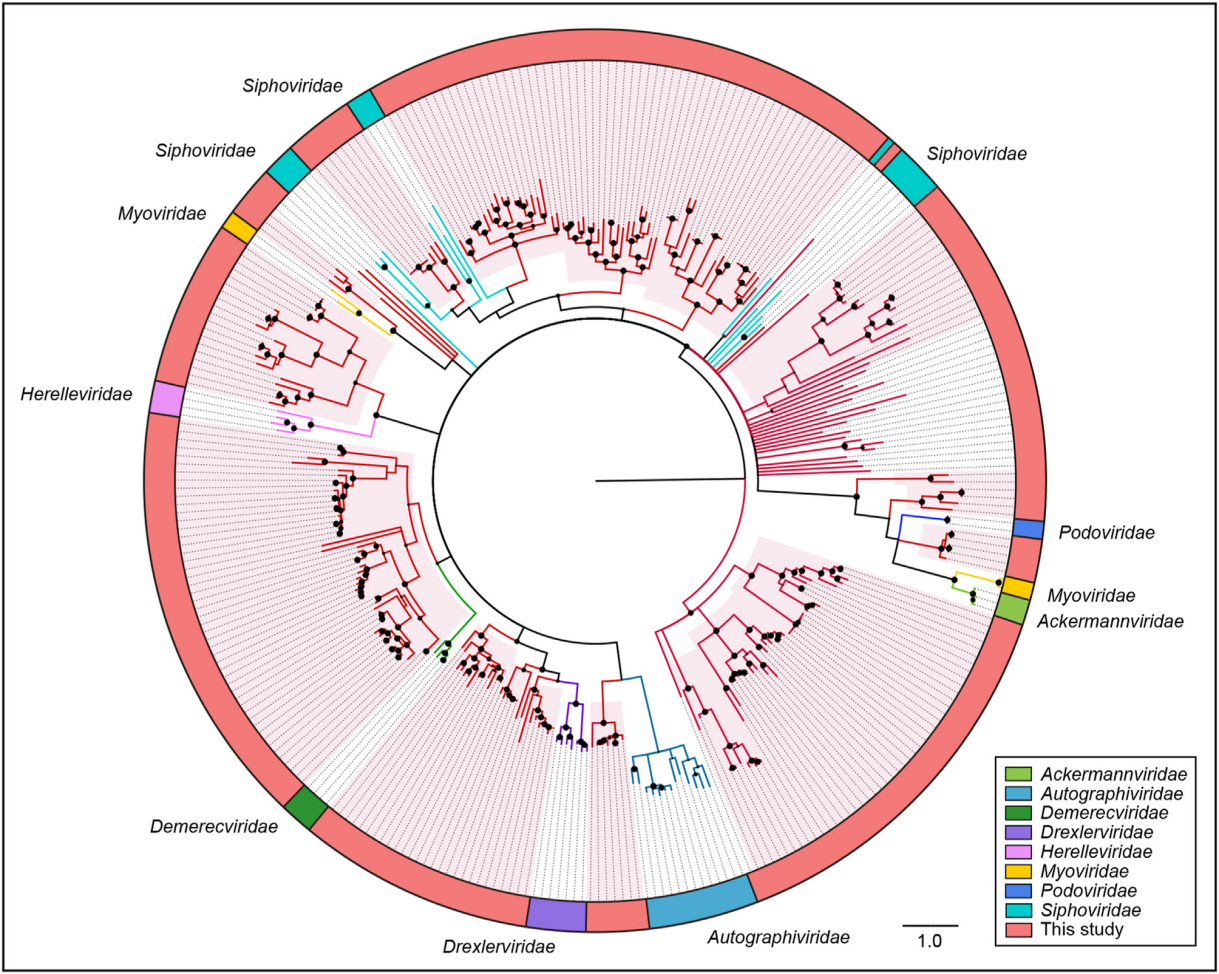
The phylogeny of *Caudovirales* identified in the Yangtze River. Bayesian inference tree was established based on amino acid sequences of TerL protein. Representative strains of all families in *Caudovirales* are included. The viruses found in this study are indicated by red lines. The red-filled sectors indicate the novel phylogenetic clusters formed by viruses unclustered with any known species. The size of the black dots on nodes is positively correlated with the corresponding bootstrap score. Reference sequences and corresponding viral families are marked with consistent colors (see color legend). The scale bar indicates the amino acid substitutions.

**Fig. 5:**
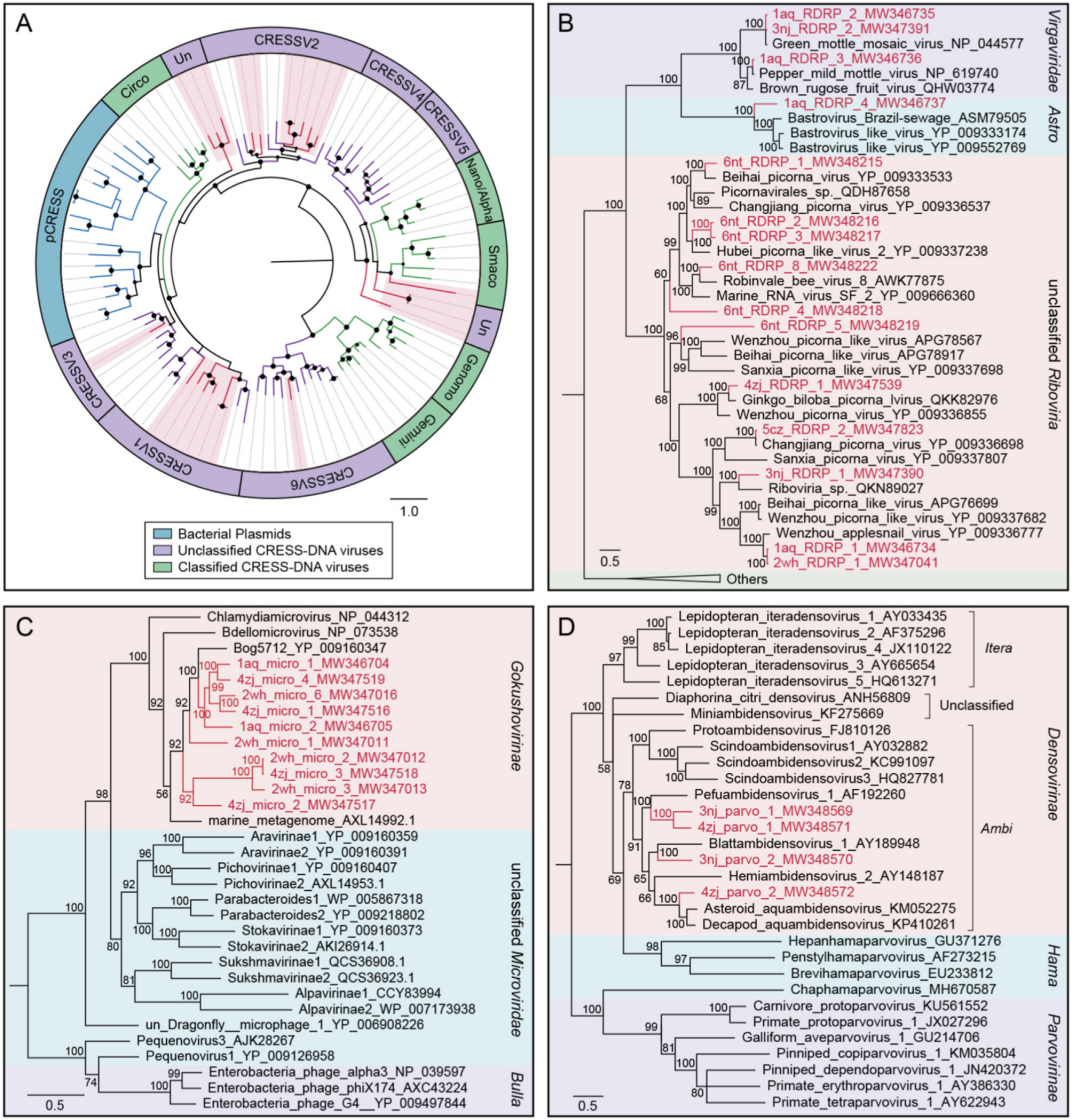
The phylogenies of ssDNA and RNA viruses identified in the Yangtze River. A Bayesian inference tree established based on amino acid sequences of Rep protein of CRESS-DNA viruses. The viruses found in this study are marked with red lines and red-filled sectors. The size of the black dots on nodes is positively correlated with the corresponding bootstrap score. Reference sequences and corresponding viral group are marked with consistent colors (see color legend). **B** Bayesian inference tree established based on amino acid sequences of RdRp protein of RNA viruses. **C** Bayesian inference tree established based on amino acid sequences of MCP of *Microviridae*. **D** Bayesian inference tree established based on amino acid sequences of NS1 protein of *Parvoviridae*. Within trees in **B**, **C**, **D**, the viruses found in this study are marked with red lines and letters. Representative strains of all genera in each family are included. Each scale bar indicates the amino acid substitutions per site. Different taxonomic clusters were represented by rectangles filled with different colors and taxon names are indicated on the right of the rectangles.

To further evaluate the commonality and diversity of the viral sequences annotated with virus hallmark genes, phylogenetic analyses were performed based on the amino acid sequences of each representatively complete marker region. Totally, 301 sequences with virus hallmark genes were selected for constructing phylogenetic trees. The order *Caudovirales* form a group of dsDNA bacteriophages with a conserved region known as large terminase subunits (TerL). The phylogenetic tree was constructed based on protein sequences of TerL (Fig. 4). The topological structure indicated that most of the 248 phage sequences were too divergent to be classified into known families within the order *Caudovirales* and formed several separate clades, demonstrating the substantial genetic diversity of tailed bacteriophages. Meanwhile, the phylogenetic tree based on 24 replication proteins (Rep) sequences also suggested the unobserved diversity of CRESS-DNA viruses. The Rep sequences all phylogenetically fell into unclassified CRESS-DNA viruses and six of them were located in known virus groups and formed several potential new unclassified groups (Fig. 5A). These results suggested the considerable unexplored viral diversity in the Yangtze River. However, in other virus groups, almost all of the selected hallmark gene sequences showed close relationship to known viral species.

Fifteen RNA virus sequences generated from six libraries were selected to perform the phylogenetic analysis based on RdRp protein sequences. Three of them were closely clustered with viral species belonging to *Virgaviridae* family which infect a variety of plants, and thereinto, one sequence (GenBank No. MW346736) shared >99% genome sequence identity with *Pepper mild mottle virus* (PMMoV), an indicator of fecal pollution in surface water (Rosario et al., 2009; Gyawali et al., 2019). Another sequence (GenBank No. MW346737) from Anqing shared about 36% protein sequence identity with known bastroviruses in *Astroviridae* family previously identified in bats, mosquitoes and sewage samples. In addition, the remaining sequences were grouped together with several known viral species belonging to unclassified *Riboviria* (Fig. 5B). Similarly, the tree based on the MCP for *Microviridae* indicated that the 10 sequences were all phylogenetically grouped into the *Gokushovirinae* lineage (Fig. 5C). And as presented in the dendrogram over NS1 for *Parvoviridae*, the four sequences were all clustered into the clade of genera *Ambidensovirus* that infect insects (Fig. 5D). These results indicated that these virus groups were relatively conservative in phylogeny.

## 4 Discussion

Aquatic ecological environments possess wide varieties of viruses that play a vital role in controlling bacterial communities and regulating biogeochemical cycles (Fischer and Velimirov, 2002; Jover et al., 2014). However, limited studies are available for the viral communities in river water systems. In the present study, we collected six water samples along different locations from the longest river in Asia, in order to acquire a vivid understanding of virome in the river water system.

The composition of viral communities in the Yangtze River displayed slight regional variations but was similar holistically. The viromes in this study were all dominated by *Caudovirales*, which consisted of *Siphoviridae*, *Podoviridae*, *Myoviridae* and other bacteriophages families. The result suggested that bacteriophages had numerical superiority in the river water system, which was consistent with the freshwater viromes in East Lake (Ge et al., 2013) and Jiulong River Estuary (Cai et al.,^2016^) in China. On the other hand, the extensive number of *Caudovirales* genomes in the phage database would in turn make it easier for query sequences to be assigned to this order. Thus, it came as no surprise that *Caudovirales* usually accounted for the largest part of identified phages. At the species level, the rarefaction curves indicated that almost all known viral species in samples were covered by deep sequencing, and the differences of species in numbers among each sample were not obvious. Totally, 201 viral species were detected in the six viral communities because only a few representatives of freshwater viruses are present in the public database, indicating that a large number of unknown viruses in river water are yet to be discovered. Besides, comparison analysis presented that 37 species were shared in the six viromes. Thereinto, a group of species *Freshwater phage uvFW* is particularly prevalent among samples. They were actinophages belonging to *Podoviridae* recovered from a reservoir in Spain, capable of modulating the Actinobacteria communities in freshwater environments (Ghai et al., 2017). The second ubiquitous species were *Rhodoferax phage* P26218 isolated from a freshwater lake in Korea, infecting the genus *Rhodoferax* that exist in a variety of water environments (Moon et al., 2015). Similarly, a related study of water virome in the Yangtze River Estuary found that bacteriophages also dominated the top 10 viral species, and the most abundant viral species was *Puniceispirillum phage HMO-2011*, infecting a bacterial genus belonging to *Proteobacteria* phylum (Wu et al., 2020).

Meanwhile, differences of viral communities among the six sampling sites can't be neglected. The abundances of allochthonous viral families with human or animal hosts living *ex situ*, such as *Astroviridae*, *Hepeviridae*, *Parvoviridae*, are sporadic across the different samples, which could be mainly explained by anthropogenic factors (Hewson et al., 2012; La Rosa et al., 2017). For example, the virome from Nanjing presented a relatively different pattern of viral community structure. The family *Parvoviridae*, instead of *Microviridae*, was the most abundant viral family of ssDNA viruses in Nanjing. And most of the unique viral species in our study were discovered from Nanjing, such as *Viltain virus* and *Hemipteran ambidensovirus* 3 that both belong to *Densovirinae* that infect arthropod hosts. Meanwhile, PCoA analysis and UPGMA tree both clearly separated Nanjing from other sampling sites, indicating Nanjing had a unique viral community signature. Water area was an important resting place for many types of arthropods such as shrimps, crabs, mosquitos and flies. Thus, the parvoviruses found in this study mainly infect invertebrates. Previous study presented that the ballast water impacted by more anthropogenic disturbances was more likely to harbour viruses belonging to *Densovirinae* subfamily (Kim et al., 2015). Thus, it is probably because Nanjing, as the only provincial capital city among the six sampling sites, is a densely populated and relatively vulnerable eco-environmental area that may be affected by municipal and industrial wastewater discharge. Therefore, Nanjing may possess unique hydrological characteristics, which could potentially have conferred unique virome composition features.

Besides, some water environmental issues should be taken into consideration. For example, a metagenomic study investigated the viruses in a river with serious fecal pollution and reported the existence of some plant, animal and human pathogenic viruses, indicating this region was facing double tasks of water harnessing and water ecological restoration (Fernandez-Cassi et al., 2017). Whereas in our study, the pathogenic enteric viruses, such as adenoviruses, enteroviruses and rotaviruses, were not detected. Moreover, *Pepper mild mottle virus* (PMMoV) is a promising indicator of fecal pollution in river water (Rosario et al., 2009; Gyawali et al., 2019). In the study, only one viral genome from Anqing shared high sequence identity with PMMoV. Other sampling sites might contain a relatively low concentration of PMMoV, leading to insufficient numbers of reads assembled into longer contigs to be phylogenetically analyzed. These phenomena suggested that the river water system in our study was mildly contaminated and the river ecological environment was in balance. Apart from fecal contamination, other water environmental problems, such as eutrophication of freshwater, also should be a matter of concern. Cyanobacterial blooms caused by eutrophication has become a serious problem threatening the safety of freshwater (Paerl and Otten, 2013). The viral community in Wuhu contained relatively high abundance of unique viral species belonging to cyanophages that infect *Cyanobacteria*. Cyanophages were able to recirculate nutrients through modulating host communities, and inhibiting the occurrence of water bloom (Paerl and Otten, 2013). Thus, this result indicated that the discharge of pollutants in the region could pose a potential influence on river water virome. However, some water ecosystems are capable of self-purification to a certain degree, and a research has indeed suggested that some microbial communities in freshwater lakes were resilient to natural and anthropogenic disturbances (Shade et al., 2012).

Virus hallmark genes refer to genes that are relatively conserved in a specific group of viruses (Koonin et al., 2006). In this study, the phylogenetic analyses based on virus hallmark genes of main virus groups revealed the diversity and stability of these virus groups. The order *Caudovirales* and CRESS-DNA viruses were both characterized by high genetic diversity, thus; sequences of the two groups were either clustered with unclassified viruses or located between these known clusters forming several new clusters. These results were generally the same as virome studies of freshwater lakes in France (Roux et al., 2012) and water ecosystems in Antarctic (Yang et al., 2019). However, unlike the aforementioned studies, sequences in *Microviridae*, *Parvoviridae* and the realm *Riboviria* in this study were all closely clustered with known viral species, suggesting a relatively conservative feature of these virus groups. For instance, *Gokushovirinae* is one of the leading subfamilies in *Microviridae* that infect certain obligate intracellular parasites and widely existed in various aquatic systems (Brentlinger et al., 2002; Roux et al., 2012; Labonte and Suttle, 2013). Viral genomes of *Microviridae* in this study were all clustered into the clade of *Gokushovirinae* and showed a close relationship to a known viral species isolated from peat soil. The results differed from the previous studies that found several distinct clusters divergent to known species in *Gokushovirinae* subfamily (Roux et al., 2012; Labonte and Suttle, 2013). In addition, the family *Parvoviridae* was rarely detected in marine water (Gregory et al., 2019) and freshwater lake (Lopez-Bueno et al., 2009; Roux et al., 2012; Yang et al., 2019) but frequently detected in waste water from human activity, such as sewage (Fernandez-Cassi et al., 2018; Martinez-Puchol et al., 2020) and ballast water (Kim et al., 2015). In the study, viromes from Nanjing and Zhenjiang possessed a relatively high abundance of reads assigned to *Parvoviridae* family. All genomes in the family were phylogenetically clustered with species in genus *Ambidensovirus* which infect a variety of insects, indicating the presence of relevant insect hosts and the potential influence of human activity.

In conclusion, our study first investigated the characteristics of viral communities in the Yangtze River. The composition of viral communities in the Yangtze River contained slightly regional variations but was similar on the whole. The virus hallmark genes presented both diverse and conservative characteristics and formed several new phylogenetic clusters. Although some limitations emanating from sample range and sequencing method inevitably existed, this study has largely enhanced our understanding of viruses in river water system, prompting further studies on the hidden diversity of viral species in a broader and deeper vision.

## Data availability

The raw sequence reads data analyzed in this study are available at the National Center for Biotechnology Information (NCBI) Sequence Read Archive database under the accession numbers SRR12904122, SRR12904128, SRR12904125, SRR12904131, SRR12904201, SRR12904457, and SRR14308507 (control ddH_2_O). All viral sequences with virus hallmark genes identified in this study were deposited in the GenBank database under the accession numbers listed in Supplementary Table S2, along with a detailed list of the viral strain names, sequence length, taxonomic classifications, etc.

## Ethics statement

This article does not contain any studies with human or animal subjects performed by any of the authors.

## Author contributions

Juan Lu: Data curation, Formal analysis, Visualization, Writing – original draft. Shixing Yang: Data curation, Formal analysis, Software, Methodology. Xiaodan Zhang: Data curation, Formal analysis, Methodology. Xiangming Tang: Data curation, Formal analysis, Resources, Methodology. Ju Zhang: Investigation, Formal analysis, Methodology. Xiaochun Wang: Investigation, Formal analysis, Methodology. Hao Wang: Supervision, Methodology, Project administration, Writing – review & editing. Quan Shen: Conceptualization, Methodology, Project administration, Writing – review & editing. Wen Zhang: Conceptualization, Methodology, Funding acquisition, Project administration, Writing – review & editing.

## Conflict of interest

The authors declare that they have no conflict of interest.
