# Supplementary material for: Metagenomic analysis of viral community in the Yangtze River expands known eukaryotic and prokaryotic virus diversity in freshwater
Source: Virol Sin. 2022 Jan 13;37(1):60–9. doi: 10.1016/j.virs.2022.01.003 (PMC8922420; doi:10.1016/j.virs.2022.01.003)
Supplement: Multimedia component 1 [file mmc1.docx]

**Virologica Sinica**

**Supplementary Data**

**Metagenomic Analysis of Viral Community in the Yangtze River Expands Known Eukaryotic and Prokaryotic Virus Diversity in Freshwater**

**Juan Lu^a,b,1^, Shixing Yang^a,1^, Xiaodan Zhang^c,1^, Xiangming Tang^d,1^, Ju Zhang^a^, Xiaochun Wang^a^, Hao Wang^b,**^, Quan Shen^a,*^, Wen Zhang^a,*^**

1. *Department of Laboratory Medicine, School of Medicine, Jiangsu University, Zhenjiang 212013, China.*
2. *Department of Clinical Laboratory, The Affiliated Huai'an Hospital of Xuzhou Medical University, Huai'an 223002, China.*
3. *Zhenjiang Center for Disease Prevention and Control, Zhenjiang 212000, China.*
4. *State Key Laboratory of Lake Science and Environment, Nanjing Institute of Geography and Limnology, Chinese Academy of Sciences, Nanjing 210008, China.*

* Corresponding author. Department of Laboratory Medicine, School of Medicine, Jiangsu University, Zhenjiang 212013, China.

** Corresponding author. Department of Clinical Laboratory, The Affiliated Huai'an Hospital of Xuzhou Medical University, Huai'an 223002, China.

E-mail addresses: z0216wen@yahoo.com (W. Zhang), shenquan@ujs.edu.cn (Q. Shen), 863251487@qq.com (H. Wang).

1 Juan Lu, Shixing Yang, Xiaodan Zhang, and Xiangming Tang contributed equally to this work.


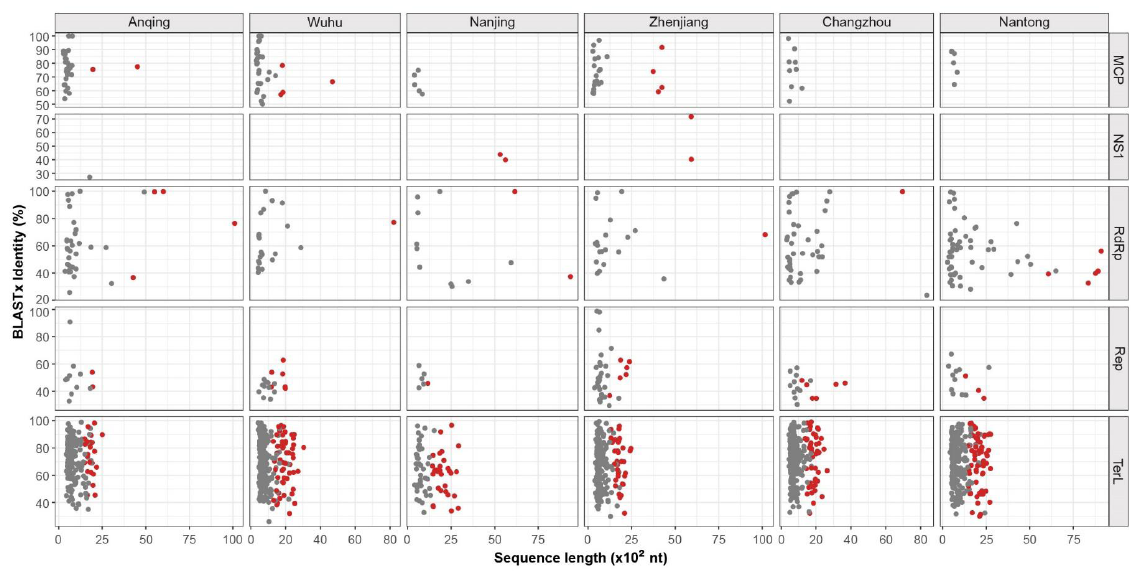


**Supplementary Fig. S1** Length and identity distribution of sequences with virus hallmark genes in the six viromes. The horizontal axis above shows sampling sites and the right vertical axis shows the types of virus hallmark genes. The horizontal axis below indicates the sequence length and the left vertical axis indicates sequence identity based on comparison between the 1,606 viral sequences in this study and their best matches in BLASTx search, respectively. Red dots represent sequences with complete CDS selected for further phylogenetic analysis, and other sequences are marked with gray dots.

**Supplementary Table S1** Information of sampling sites and corresponding libraries.


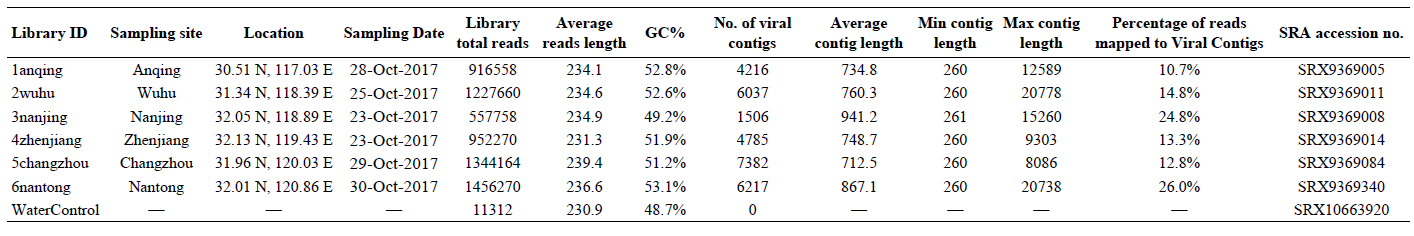


**Supplementary Table S2** Information of viral sequences with virus hallmark genes identified in the Yangtze River.

(Please see separate excel file.)
